# Supplementary material for: Goal-oriented representations in the human hippocampus during planning and navigation
Source: Nat Commun. 2023 May 23;14:2946. doi: 10.1038/s41467-023-35967-6 (PMC10206082; doi:10.1038/s41467-023-35967-6)
Supplement: Supplementary file 1 — Supplementary Information [file 41467_2023_35967_MOESM1_ESM.pdf]

Supplemental Materials

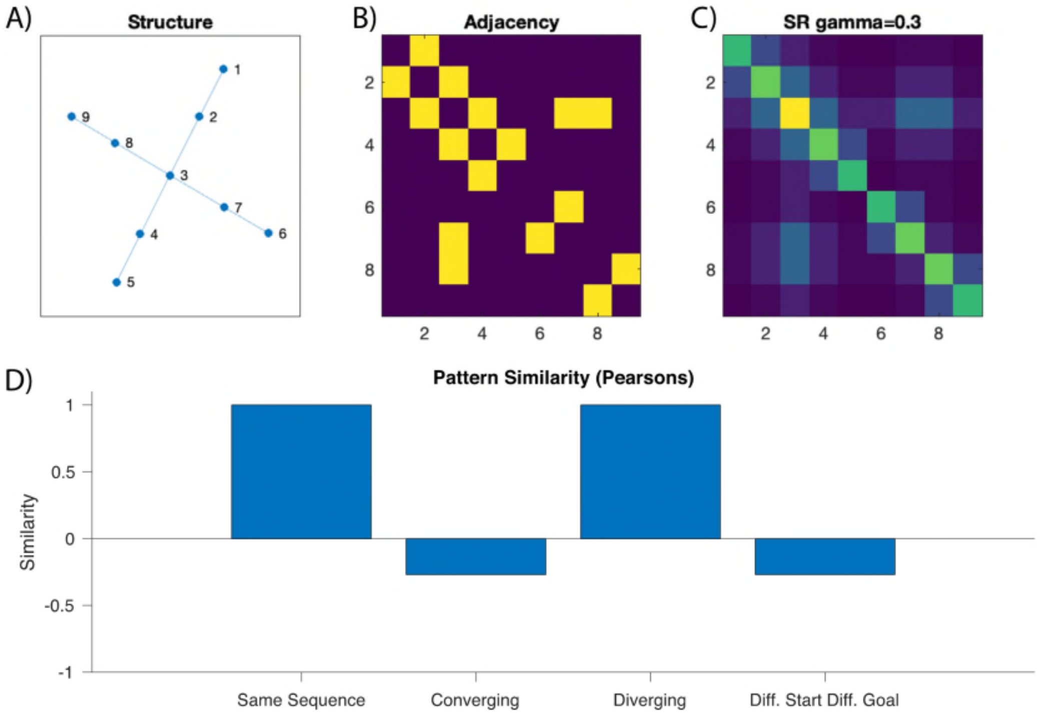

Supplemental Figure 1 - Simulation of Successor Representation (SR) model

Supplemental Figure 1 - Simulation of Successor Representation (SR) model

A) Topological structure of states used in simulation. Numbers identify individual states. Structure is identical to a single zoo context condition used in our experiment. B) Adjacency matrix of the topological structure. C) SR Matrix using Gamma of 0.3. D) Pattern similarity results. We tested the hypothesis that during planning the hippocampus encodes an SR representation of the first position in the sequence. We simulated three sequences state 1 -> state 5, state 6 -> state 5, and state 1-> state 9. We then indexed the rows of the SR matrix corresponding to the first position in each planned sequence and calculated pairwise similarity using pearsons. Same Sequence = Same Sequence = 1->5 cor 1->5; Converging = 6->5 cor 1->5; Diverging = 1->9 cor 1->5; Diff. Start Diff. Goal 6->5 cor 1->9.

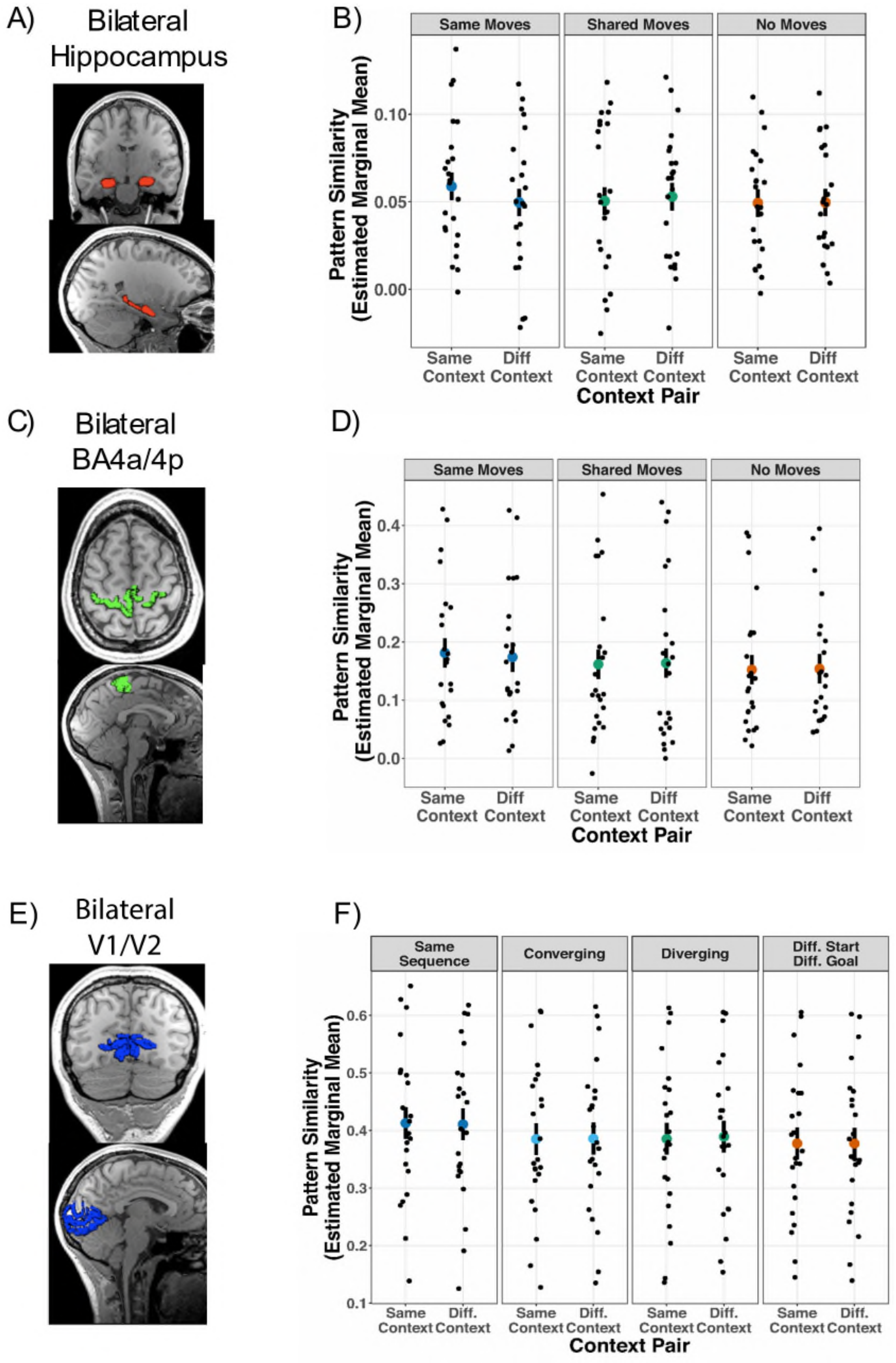

Supplemental Figure 2 - Control analyses in Visual and Motor ROIs

Supplemental Figure 2 - Control analyses in Visual and Motor ROIs

A) Bilateral hippocampal ROI on a representative participant's anatomical scan

B) Control analysis contrasting the effect of shared planned motor information on hippocampal representations. Results showed no effect of planned moves or context on pattern similarity (main effect of context: $\chi^2(1, N = 23) = 0.46$, $p = 0.5$; main effect of move: $\chi^2(2, N = 23) = 1.56$, $p = 0.46$; interaction: $\chi^2(2, N = 23) = 2.68$, $p = 0.26$). C) Bilateral primary motor ROI on representative participant's anatomical scan. D) Same as B but examining BA4a/p. Importantly this analysis showed that planned movement was not modulated by context (main effect of move: $\chi^2(2, N = 23) = 13.95$, $p < 0.001$; main effect of context: $\chi^2(1, N = 23)$, $= 0.06$, $p = 0.81$; interaction: $\chi^2(2, N = 23)$, $= 0.68$, $p = 0.71$). E) Bilateral primary visual ROI on representative participant's anatomical scan.

F) Control analysis in V1/V2 ROI depicting that visual stimuli is not modulated by context (main effect of overlap: $\chi^2(3, N = 23\ ) = 90.24$, $p < 0.001$; main effect of context: $\chi^2(1\ N = 23\ ) = 0.05$; interaction: $p = 0.82$; $\chi^2(3, N = 23\ ) = 0.76$, $p = 0.86$.

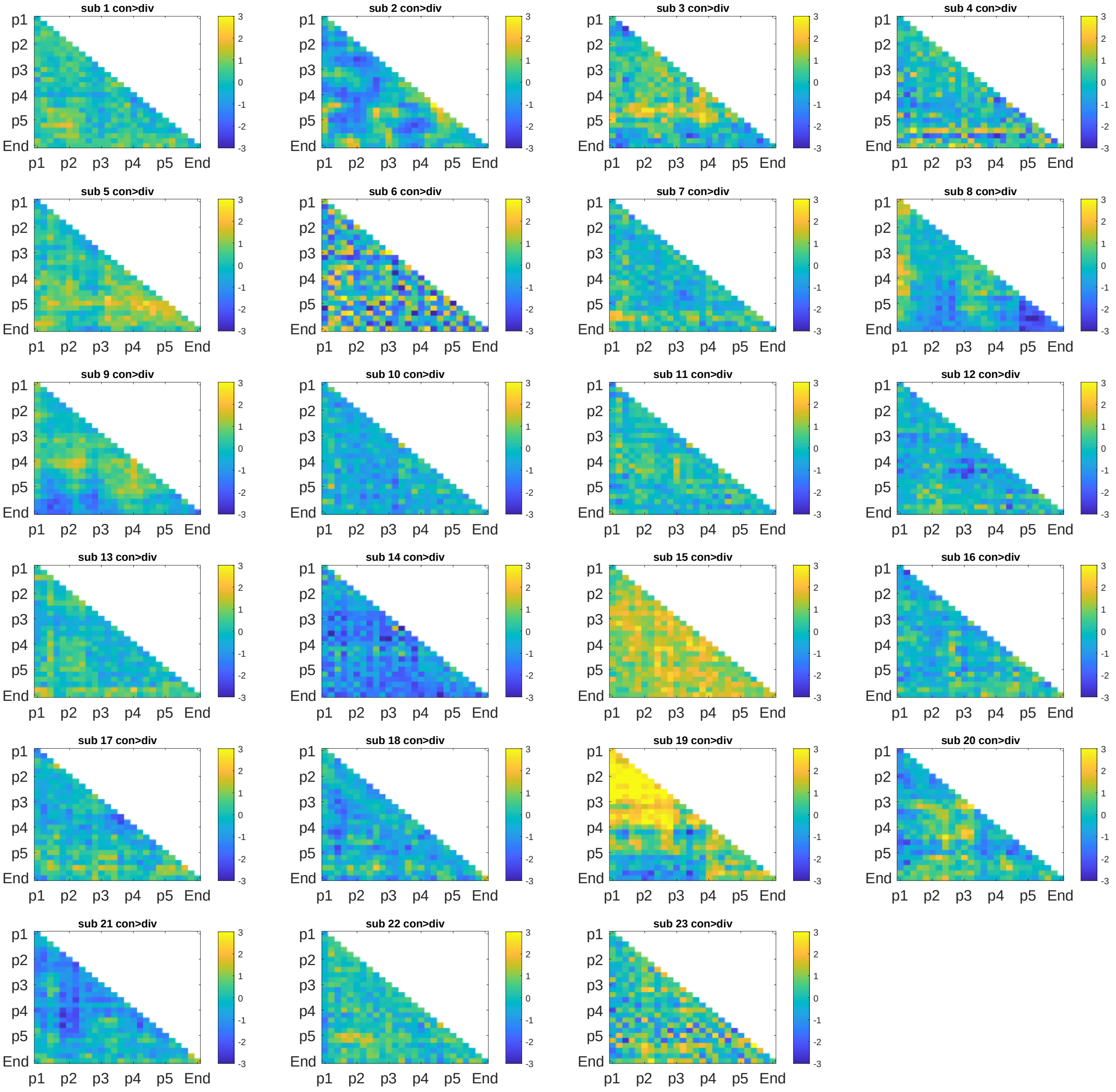

Supplemental Figure 3 - Single participant TR by TR similarity plots for converging > diverging in the same context from bilateral hippocampus

Supplemental Figure 3 - Single participant TR by TR similarity plots for converging > diverging in the same context from bilateral hippocampus

Single participant TR by TR similarity plots for converging > diverging in the same context from bilateral hippocampus. Plots are Z scored across participants for each plot to preserve relative scaling between plots.

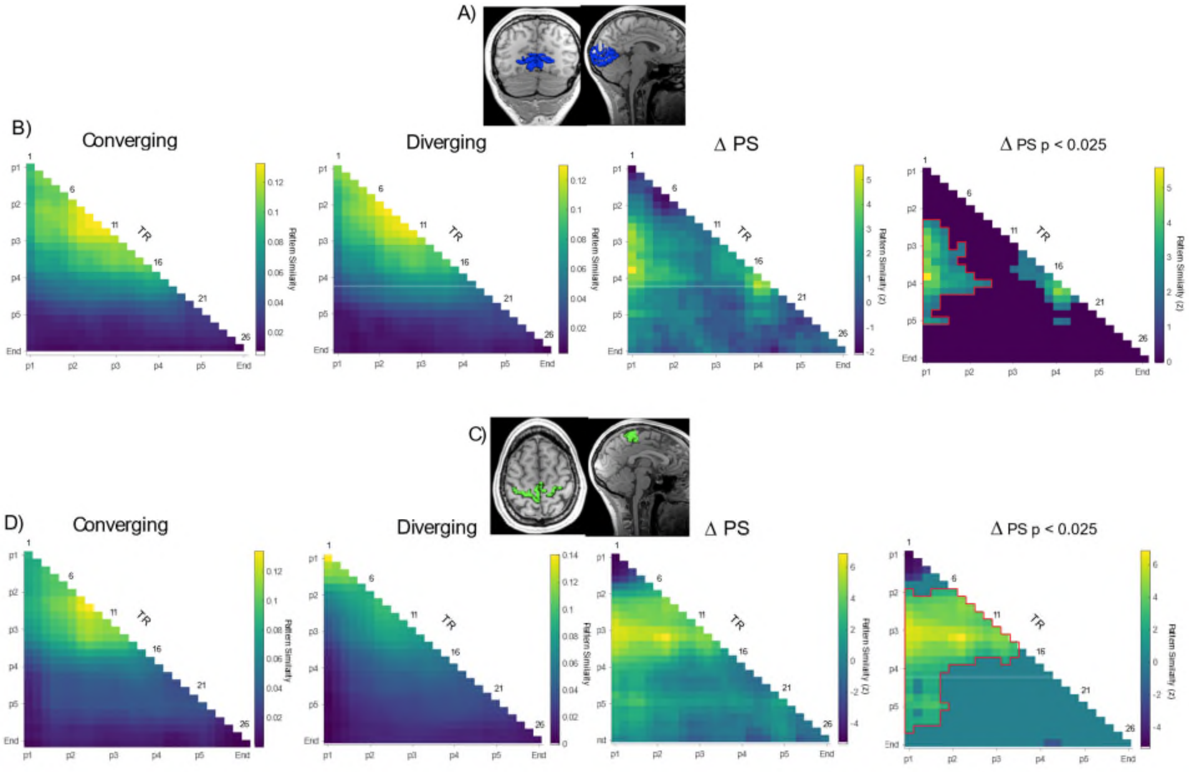

Supplemental Figure 4 – Visual and Motor regions show off-diagonal reinstatement of upcoming visual and action information

Supplemental Figure 4 – Visual and Motor regions show off-diagonal reinstatement of upcoming visual and action information

A) Bilateral V1/V2 ROI displayed on representative participant's anatomical scan B) Group level pattern similarity results from converging and diverging sequences during active navigation. ∆PS: TR by TR pattern similarity results depicting a statistical map of converging – diverging. Z values were calculated using a bootstrap shuffling procedure with 10,000 permutations. Thresholded statistical map at $p < 0.025$. Cluster based permutation tests with 10,000 permutations (Maris and Oostenveld, 2007) were performed with a cluster defining threshold of $p < 0.025$ (two-tailed) and a cluster alpha of 0.05. Outlined in red is a significant cluster of timepoints that survives multiple comparisons correction (cluster mass = 348.23, $p = 0.0052$, maximum cluster corrected) C) Bilateral BA4a/p ROI displayed on representative participant's anatomical scan. D) Same as B but in BA4a/p. Outlined in red is a significant cluster of timepoints that survives multiple comparisons correction (cluster mass = 673.79, $p < 0.0001$, maximum cluster corrected).

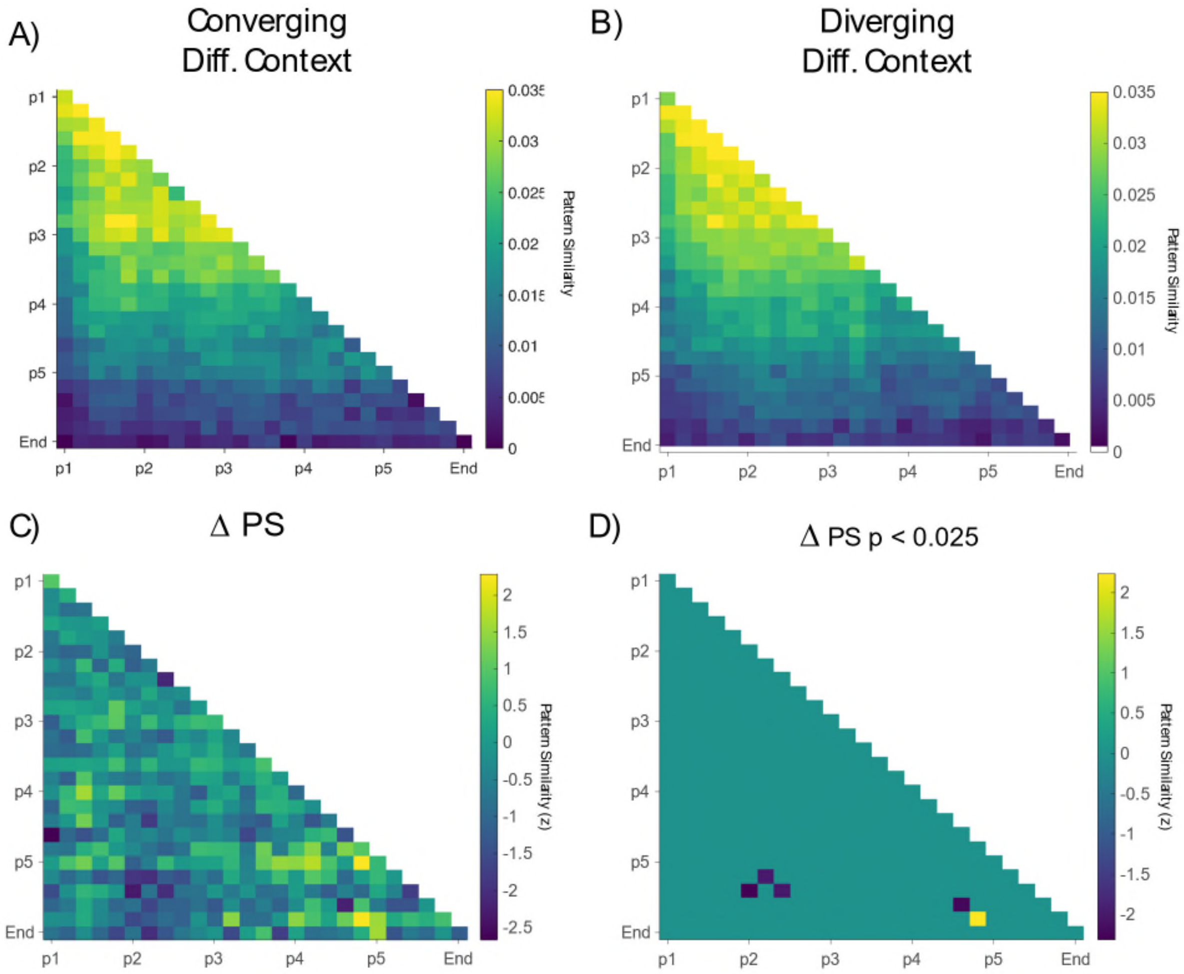

Supplemental Figure 5 – No evidence of off-diagonal reactivation for converging > diverging in different contexts

Supplemental Figure 5 – No evidence of off-diagonal reactivation for converging > diverging in different contexts

A) Group level pattern similarity results from converging sequences in different context during active navigation. B) Same as A) but showing diverging sequences in different contexts. C) TR by TR pattern similarity results depicting a statistical map of converging – diverging. Z values were calculated using a bootstrap shuffling procedure with 10,000 permutations. D) Thresholded statistical map at $p < 0.025$ (two-tailed). Cluster based permutation tests with 10,000 permutations (Maris and Oostenveld, 2007) were performed with a cluster defining threshold of $p < 0.025$ and a cluster alpha of 0.05 (two-tailed, maximum cluster corrected). Note that no clusters survive multiple comparisons correction.

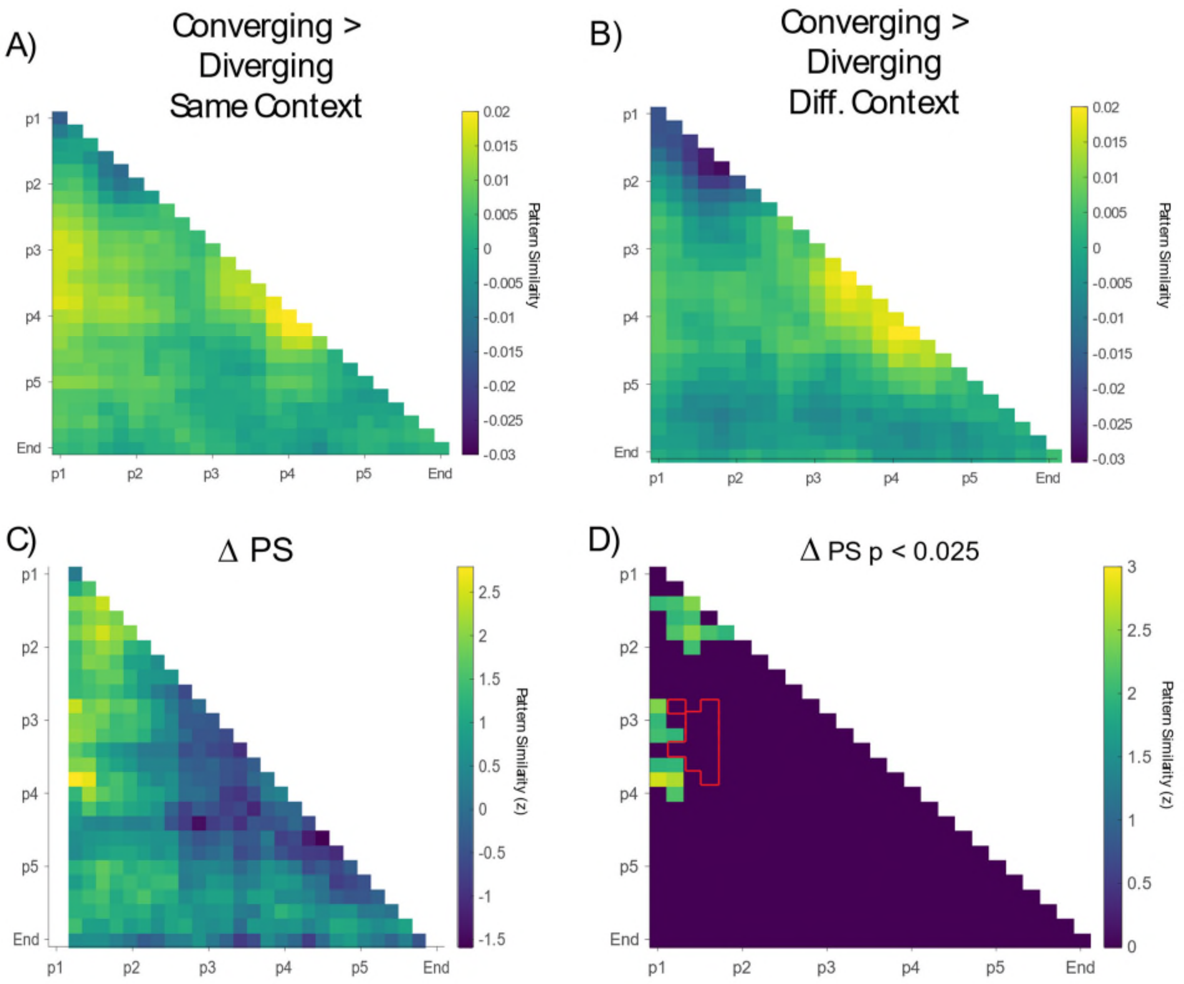

Supplemental Figure 6 – Context interaction for Converging > Diverging

Supplemental Figure 6 – Context interaction for Converging > Diverging

A) Group level pattern similarity results from converging sequences > diverging sequences in the same context during active navigation. B) Same as A) but showing converging > diverging sequences in different contexts. C) TR by TR pattern similarity results depicting a statistical map of the interaction effect (converging > diverging same context) – (converging > diverging diff. context). Z values were calculated using a bootstrap shuffling procedure with 10,000 permutations. D) Thresholded statistical map at $p < 0.025$ (two-tailed). Cluster based permutation tests with 10,000 permutations were performed with a cluster defining threshold of $p < 0.025$ and a cluster alpha of 0.05 (two-tailed, maximum cluster corrected). Note that no clusters survive multiple comparisons. Outlined in Red is the cluster extent of the cluster identified in Converging > Diverging Same Context from Figure 4d in the manuscript.

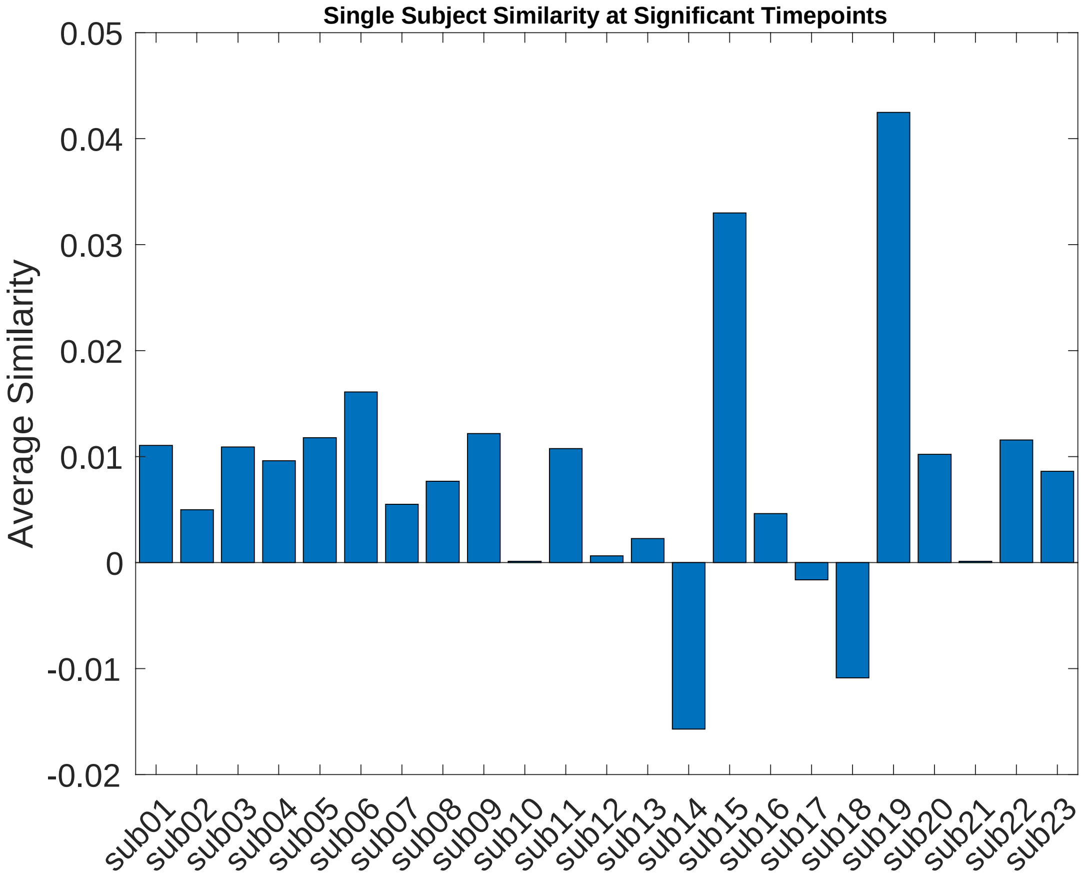

Supplemental Figure 7 – Single participant mean pattern similarity at significant timepoints from bilateral hippocampus

Supplemental Figure 7 – Single participant mean pattern similarity at significant timepoints from bilateral hippocampus

Average converging > diverging effect from individual participants in bilateral hippocampus from cluster identified in Figure 4d (outlined in red). Pattern similarity was obtained by averaging pattern similarity values from pixels contained within the significant group level cluster (14 pixels). Note that 20 of 23 participants showed the group level effect. Formally, this was confirmed using a Wilcoxon signed rank test on the similarity values obtained from the procedure outlined above. This revealed that a majority of participants showed an increase in pattern similarity during these timepoints (Signed Rank = 238, Z = 3.042, p = 0.0024).

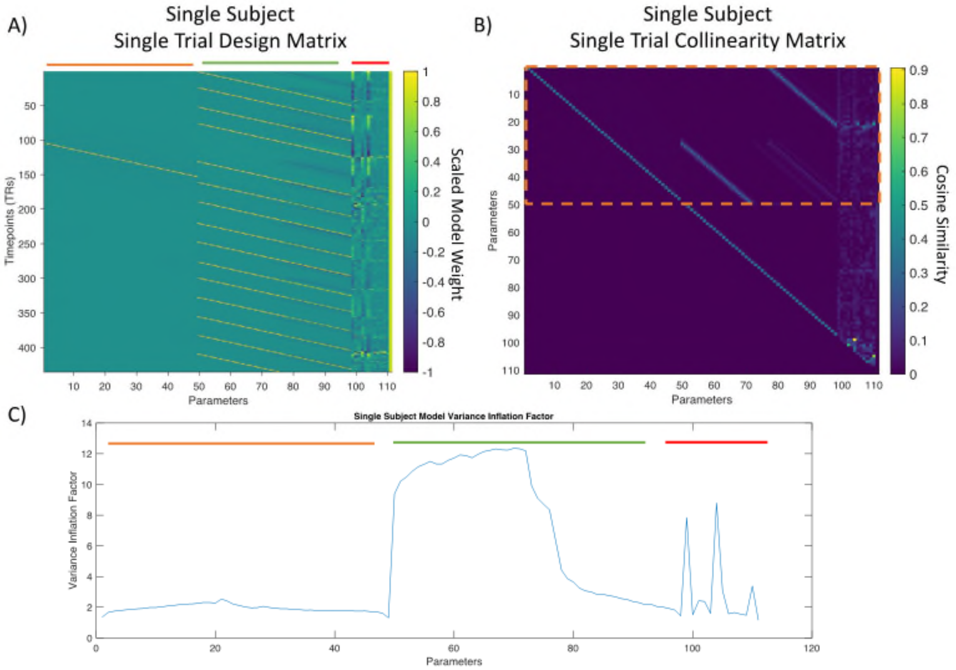

Supplemental Figure 8 – Single participant design matrix and model diagnostics for FIR modelling approach.

Supplemental Figure 8 – Single participant design matrix and model diagnostics for FIR modelling approach.

A) A single participant design matrix for one sequence modelled with our FIR approach. The orange, green and red bars highlight parameters associated with current sequence being modelled, nuisance regressors, and motion parameters, respectively. B) A parameter-by-parameter collinearity matrix measured with cosine similarity. The lower left and diagonal elements have been excluded to aid in visualization. Values of 0 illustrate orthogonal regressors whereas values closer to 1 illustrate a degree of collinearity. The orange box outlines the rows of the matrix that are associated with the sequence being modeled. C) Variance inflation factor as a function of individual parameters. The orange bar is intended to direct the reader to timepoints that are used in RSA. Note that parameters associated with the sequence being modeled have relatively stable VIF and are within commonly held standards within the literature (Mumford et al., 2015).

Supplemental Table 1 – Results from linear mixed effects model testing for a sequence by context interaction using likelihood ratio tests

DF full model: 6

Effect	DF	Chi Sq.	P Val.
Sequence	1	3.57	0.059
Context	1	3.36	0.067
Sequence * Context	1	4.26	0.039

Supplemental Table 2 - Results from linear mixed effects model testing for a sequence by context interaction using F-Tests

Effect	DF	F	P Val.
Sequence	1,66	3.5	0.066
Context	1,66	3.3	0.074
Sequence * Context	1,66	4.2	0.044

Supplemental Table 3 - Results from linear mixed effects model testing for an overlap by context interaction using likelihood ratio tests

DF full model: 10

Effect	DF	Chi Sq	P Val.
Context	1	2.03	0.15
Overlap	3	4.85	0.18
Context * Overlap	3	14.75	0.002

Supplemental Table 4 - Results from linear mixed effects model testing for an overlap by context interaction using F-Tests

Effect	DF	F	P Val.
Context	1,154	1.95	0.16
Overlap	3,154	1.57	0.2
Context * Overlap	3,154	4.93	0.003

Supplemental Tables – Comparison of statistical test used for assessing significance in mixed models. Note that the use of Chi Squared tests vs. F-Tests do not impact our results or conclusions. F-Tests were conducted using two-tailed tests.

Supplementary References

1. Maris, E., & Oostenveld, R. Nonparametric statistical testing of EEG- and MEG-data. *Journal of Neuroscience Methods*, *164*(1), 177–190. https://doi.org/10.1016/j.jneumeth.2007.03.024 (2007).

2. Mumford, J. A., Poline, J. B., & Poldrack, R. A. Orthogonalization of regressors in fMRI models. *PLoS ONE*, *10*(4), 1–11. https://doi.org/10.1371/journal.pone.0126255 (2015).
