# Supplementary material for: Goal-oriented representations in the human hippocampus during planning and navigation
Source: Nat Commun. 2023 May 23;14:2946. doi: 10.1038/s41467-023-35967-6 (PMC10206082; doi:10.1038/s41467-023-35967-6)
Supplement: Supplementary file 3 — Reporting Summary [file 41467_2023_35967_MOESM3_ESM.pdf]

nature portfolio

Corresponding author(s): Jordan Crivelli-Decker

Last updated by author(s): 1/7/2023

Reporting Summary

Nature Portfolio wishes to improve the reproducibility of the work that we publish. This form provides structure for consistency and transparency in reporting. For further information on Nature Portfolio policies, see our Editorial Policies and the Editorial Policy Checklist.

Statistics

For all statistical analyses, confirm that the following items are present in the figure legend, table legend, main text, or Methods section.

n/a	Confirmed	
☐	☒	The exact sample size (*n*) for each experimental group/condition, given as a discrete number and unit of measurement
☐	☒	A statement on whether measurements were taken from distinct samples or whether the same sample was measured repeatedly
☐	☒	The statistical test(s) used AND whether they are one- or two-sided<br>*Only common tests should be described solely by name; describe more complex techniques in the Methods section.*
☐	☒	A description of all covariates tested
☐	☒	A description of any assumptions or corrections, such as tests of normality and adjustment for multiple comparisons
☐	☒	A full description of the statistical parameters including central tendency (e.g. means) or other basic estimates (e.g. regression coefficient) AND variation (e.g. standard deviation) or associated estimates of uncertainty (e.g. confidence intervals)
☐	☒	For null hypothesis testing, the test statistic (e.g. *F*, *t*, *r*) with confidence intervals, effect sizes, degrees of freedom and *P* value noted<br>*Give P values as exact values whenever suitable.*
☒	☐	For Bayesian analysis, information on the choice of priors and Markov chain Monte Carlo settings
☐	☒	For hierarchical and complex designs, identification of the appropriate level for tests and full reporting of outcomes
☐	☒	Estimates of effect sizes (e.g. Cohen's *d*, Pearson's *r*), indicating how they were calculated

Our web collection on statistics for biologists contains articles on many of the points above.

Software and code

Policy information about availability of computer code

Data collection	Matlab 2016a, Psychophysics toolbox (3.0.18.12)
Data analysis	Matlab 2016a, R 3.6.0, Python 3.6, lme4 (1.1-26), SPM12, emmeans (1.5.5-1), afex (0.28-1) , freesurfer (v7.2.0), all custom code to reproduce analyses available at https://github.com/jecd/Hippocampgoal

For manuscripts utilizing custom algorithms or software that are central to the research but not yet described in published literature, software must be made available to editors and reviewers. We strongly encourage code deposition in a community repository (e.g. GitHub). See the Nature Portfolio guidelines for submitting code & software for further information.

Data

Policy information about availability of data

All manuscripts must include a data availability statement. This statement should provide the following information, where applicable:

- Accession codes, unique identifiers, or web links for publicly available datasets
- A description of any restrictions on data availability
- For clinical datasets or third party data, please ensure that the statement adheres to our policy

All preprocessed data for this study are available at: https://github.com/jecd/Hippocampgoal. Raw data are available in an OSF repository: https://osf.io/txauh/.

Human research participants

Policy information about studies involving human research participants and Sex and Gender in Research.

Reporting on sex and gender	11 people identifying as male and 12 people identifying as female participated in this study. We do not believe gender effects impact the results reported here.
Population characteristics	18-25 year old human UC Davis graduate and undergraduate students. 11 Male and 12 female. All right-handed and native English speakers.
Recruitment	Participants were recruited from UC Davis' paid subject pool and from the UC Davis Psychology graduate group. Self-selection for highly motivated participants is likely due to difficulties maintaining a paid participant account. This likely impacted behavioral performance positively.
Ethics oversight	Institutional Review Board at UC Davis

Note that full information on the approval of the study protocol must also be provided in the manuscript.

Field-specific reporting

Please select the one below that is the best fit for your research. If you are not sure, read the appropriate sections before making your selection.

☐ Life sciences ☒ Behavioural & social sciences ☐ Ecological, evolutionary & environmental sciences

For a reference copy of the document with all sections, see nature.com/documents/nr-reporting-summary-flat.pdf

Behavioural & social sciences study design

All studies must disclose on these points even when the disclosure is negative.

Study description	Within subjects design evaluating the effect of context and goals on planning representations in the brain. Data are quantitative measurements of behavioral responses to stimuli and Blood Oxygen Level Dependent (BOLD) measurements during the task.
Research sample	Data were collected from healthy UC Davis undergraduates and UC Davis Graduate Students (11 male, 12 female). Age range 18-25. This sample was chosen because it is representative of the UC Davis student population.
Sampling strategy	Convenience. No sample size calculations were performed. 30 subjects were collected based on standard sample sizes for neuroimaging studies in the literature.
Data collection	Data were collected on standard windows desktop computer both inside and outside the imaging device. During imaging and behavioral testing participants were alone in the room while they performed the task. Researchers were nearby. The researcher was not blinded to the hypothesis.
Timing	September 2015 - June 2017
Data exclusions	Data from one participant was excluded due to technical complications with the fMRI scanner, one participant was excluded due to a stimulus computer malfunction, two participants were excluded due to poor behavioral performance in the scanner (defined as falling below trained criterion, 85% correct, in the scanner), and one participant was removed from the scanner before the experiment concluded because they did not wish to continue in the study. Prior to data analysis, to ensure data quality, we conducted a univariate analysis to look at motor and visual activation during the task compared to an implicit baseline (unmodeled timepoints when the participant was viewing a fixation cross). Two subjects showed little to no activation in these regions and were excluded from further analysis. The remaining 23 participants (11 male, 12 female, all right handed) are reported here.
Non-participation	One participant was removed from the scanner before the experiment concluded because they did not wish to continue in the study
Randomization	Counter-balanced order during learning of experimental stimuli (e.g. learning about Context 1 vs. Context 2 first). During testing in the scanner, order of stimuli were pseudo- randomized to ensure that trial types did not occur consecutively. Order of the experimental conditions were also counter-balanced across participants (e.g. Context 1 vs. Context 2 occurring first within a block). Participants were assigned to a counter balance group via alternating enrollment, such that sequential participants were in different counter balance groups.

Reporting for specific materials, systems and methods

We require information from authors about some types of materials, experimental systems and methods used in many studies. Here, indicate whether each material, system or method listed is relevant to your study. If you are not sure if a list item applies to your research, read the appropriate section before selecting a response.

Materials & experimental systems

n/a	Involved in the study
☒	☐ Antibodies
☒	☐ Eukaryotic cell lines
☒	☐ Palaeontology and archaeology
☒	☐ Animals and other organisms
☒	☐ Clinical data
☒	☐ Dual use research of concern

Methods

n/a	Involved in the study
☒	☐ ChIP-seq
☒	☐ Flow cytometry
☐	☒ MRI-based neuroimaging

Magnetic resonance imaging

Experimental design

Design type	Task, rapid-event related.
Design specifications	6 blocks and 72 sequences presented per subject. Each item in a sequence was presented for 3s. Inter-stimulus interval was 2s.
Behavioral performance measures	Response time and correct button press. Accuracy and reaction time were used to establish that subjects were performing the task. Mean and standard deviations were used to establish that subjects were accurate above a pre-specified accuracy criterion of 85%.

Acquisition

Imaging type(s)	Functional and structural
Field strength	3T
Sequence & imaging parameters	MRI data were acquired on a 3T Siemens Skyra MRI using a 32-channel head coil. Anatomical images were collected using a T1-weighted magnetization prepared rapid acquisition gradient echo (MP-RAGE) pulse sequence image (FOV = 256 mm; TR = 1800 ms; TE = 2.96 ms; image matrix = 256 x 256; 208 axial slices; voxel size = 1mm isotropic). Functional images were collected with a multi-band gradient echo planar imaging sequence (TR = 1222 ms; TE = 24 ms; flip angle = 67 degrees; matrix=64x64, FOV=192mm; multi-band factor = 2; 3 mm3 isotropic spatial resolution).
Area of acquisition	Whole-brain

Diffusion MRI ☐ Used ☒ Not used

Preprocessing

Preprocessing software	Matlab 2016a, SPM12, ART Repair, Freesurfer, Advanced Normalization Tools (ANTs), R, lme4. Functional data were smoothed with a 4mm FWHM 3d gaussian kernel.
Normalization	Multivariate analyses were performed in native space
Normalization template	Multivariate analyses were performed in native space
Noise and artifact removal	6 motion paramaters + movement spike regressors
Volume censoring	Spike regressors (> 0.5mm)

Statistical modeling & inference

Model type and settings	RSA
Effect(s) tested	Factorial design

Specify type of analysis: ☐ Whole brain ☒ ROI-based ☐ Both

ROI definitions were generated using a combination of Freesurfer, and a multistudy group template of the medial temporal lobe. The multistudy group template was used to generate probabilistic maps of hippocampal head, body, and tail as defined by Yushkevich et al. and warped to MNI space using Diffeomorphic Anatomical Registration Using Exponentiated Lie Algebra (DARTEL) in SPM8. Maps were created by taking the average of 55 manually-segmented ROIs and therefore reflect the likelihood that a given voxel was labeled in a participant. Masks were created by thresholding the maps at 0.5, (i.e., that

Anatomical location(s)

voxel was labeled in 50% of participants). These maps were then reverse normalized to native space using Advanced Normalization Tools (ANTS). Participant specific cortical ROIs were generated using Freesurfer version 6.0. from the Destrieux and Desikan atlas. Individual cortical ROIs were binarized and aligned to participants' native space by applying the affine transformation parameters obtained during coregistration. These masks were combined into merged masks that encompassed the entire hippocampus bilaterally (see cue period pattern similarity for more information). Anatomical ROIs for V1/V2 and BA4a/p were obtained by running all participants structural scans through the freesurfer recon-all pipeline. Our V1/V2 ROI was obtained by merging the anatomical masks for BA17 and BA18.

Statistic type for inference
(See Eklund et al. 2016)

No univariate analyses were reported.

Correction

Cluster based permutation tests with Monte Carlo simulations

Models & analysis

n/a	Involved in the study
☒	☐ Functional and/or effective connectivity
☒	☐ Graph analysis
☐	☒ Multivariate modeling or predictive analysis

Multivariate modeling and predictive analysis

Cue period pattern similarity analysis: Our primary interest was to investigate how prospective sequence representations were modulated based on context membership. To achieve this, we used representational similarity analysis to analyze multi-voxel activity patterns (Kriegeskorte et al., 2008) within regions of interest. Generalized Linear Models (GLMs) were used to obtain single trial parameter estimates of the cue period using a modified least-squares all (LSA) model (Mumford et al., 2012, Brown et al., 2016). Data were high-pass filtered using a 128s cutoff and pre-whitened using AR(1) in SPM. All events were convolved with a canonical HRF to be consistent with prior work (Mumford et al., 2012). Cue periods were modeled using separate single trial regressors for each cue (2s boxcar). The remaining portions of the task were modelled as follows: Navigation periods were modelled with separate 25s boxcar functions for each trial, separate single trial regressors for catch sequences modelled as a 15s boxcar, separate single trial catch blank trials (stick function), outcome correct at condition level (stick), outcome incorrect at condition level (stick), and the four button presses at the condition level (stick). Nuisance regressors for motion spikes, 12 motion regressors (6 for realignment and 6 for the derivatives of each of the realignment parameters) and a drift term were included in the GLM. Pattern similarity between the resulting beta images were calculated using Pearson's correlation coefficient between all pairs of trials in the experiment. Only between run trial pairs were included in the analysis to avoid spurious correlations driven by auto-correlated noise (Mumford et al., 2014). Single trial activity patterns were inputted into a linear mixed effect model to account for individual differences in pattern similarity estimates. Sequence conditions were as follows - Same Sequence, Different Sequence, Same Context, Different Context, Converging Sequence, Diverging Sequence, No Overlap.
